# Platelet lysate as a novel serum-free media supplement for the culture of equine bone marrow-derived mesenchymal stem cells

**DOI:** 10.1186/s13287-018-0823-3

**Published:** 2018-03-22

**Authors:** Maria C. Naskou, Scarlett M. Sumner, Anna Chocallo, Hannah Kemelmakher, Merrilee Thoresen, Ian Copland, Jacques Galipeau, John F. Peroni

**Affiliations:** 1 Department of Large Animal Medicine, Veterinary Medical Center, College of Veterinary Medicine, University of Georgia, 2200 College Station Road, Athens, GA 30602 USA; 2 Emory Personalized Immunotherapy Center [EPIC], Emory University School of Medicine, 100 Woodruff Circle, Atlanta, GA 30322 USA; 3 Department of Medicine and Carbone Comprehensive Cancer Center, University of Wisconsin, 600 Highland Ave., Madison, WI 53792 USA

**Keywords:** Equine platelet apheresis, Equine platelet lysate, Mesenchymal stem cells, Fetal bovine serum, Cell culture

## Abstract

**Background:**

Mesenchymal stem cells (MSCs) produced for clinical purposes rely on culture media containing fetal bovine serum (FBS) which is xenogeneic and has the potential to significantly alter the MSC phenotype, rendering these cells immunogenic. As a result of bovine-derived exogenous proteins expressed on the cell surface, MSCs may be recognized by the host immune system as non-self and be rejected. Platelet lysate (PL) may obviate some of these concerns and shows promising results in human medicine as a possible alternative to FBS. Our goal was to evaluate the use of equine platelet lysate (ePL) pooled from donor horses in place of FBS to culture equine MSCs. We hypothesized that ePL, produced following apheresis, will function as the sole media supplement to accelerate the expansion of equine bone marrow-derived MSCs without altering their phenotype and their immunomodulatory capacity.

**Methods:**

Platelet concentrate was obtained via plateletpheresis and ePL were produced via freeze-thaw and centrifugation cycles. Population doublings (PD) and doubling time (DT) of bone marrow-derived MSCs (*n* = 3) cultured with FBS or ePL media were calculated. Cell viability, immunophenotypic analysis, and trilineage differentiation capacity of MSCs were assessed accordingly. To assess the ability of MSCs to modulate inflammatory responses, *E. coli* lipopolysaccharide (LPS)-stimulated monocytes were cocultured with MSCs cultured in the two different media formulations, and cell culture supernatants were assayed for the production of tumor necrosis factor (TNF)-α.

**Results:**

Our results showed that MSCs cultured in ePL media exhibited similar proliferation rates (PD and DT) compared with those cultured in FBS at individual time points. MSCs cultured in ePL showed a statistically significant increased viability following a single washing step, expressed similar levels of MSC markers compared to FBS, and were able to differentiate towards the three lineages. Finally, MSCs cultured in ePL efficiently suppressed the release of TNF-α when exposed to LPS-stimulated monocytes similar to those cultured in FBS.

**Conclusion:**

ePL has the potential to be used for the expansion of MSCs before clinical application, avoiding the concerns associated with the use of FBS.

## Background

Mesenchymal stem cells (MSCs) are multipotent self-renewing cells that have been implicated in orchestrating the repair of damaged tissues by modulating the endogenous repair process through interacting with the inflammatory response of the injured tissue [1–3]. The preparation of MSCs before clinical application requires their primary isolation and ex vivo expansion to propagate an adequate number of cells for transplantation. Fetal bovine serum (FBS) is the current gold standard culture additive used as a source of growth factors, hormones, and vital nutrients to support MSC expansion in the laboratory [4–9]. Unfortunately, there is concerning evidence to show that FBS contains endotoxins (such as lipopolysaccharide (LPS)) and xenogeneic antigens that may alter the phenotype of MSCs grown in FBS, rendering these cells immunogenic [10–12]. This may prompt the immune system to reject MSCs following introduction into the recipient, even when the delivered MSCs are autologous to the host. The transplantation of MSCs cultured with traditional culture techniques is also a potential route of transmission of FBS-derived animal pathogens, such as prions and viruses [13–15]. Furthermore, the Food and Drug Administration (FDA) has encouraged the use of xenoprotein-free culture conditions for the expansion of MSCs in humans to avoid adverse effects related to FBS [16]. These facts, together with the rising cost of FBS and ethical concerns related to the manufacturing of FBS, underpin the rationale behind the development of FBS-free media to support the expansion of MSCs for clinical purposes.

To this end, several studies have investigated the use of platelet-derived products, such as platelet lysate (PL), obtained following the lysis of platelets from platelet concentrates or platelet-rich plasma (PRP) as a media supplement for the in vitro culture of various types of cells [4]. Human PL is a reportedly superior alternative to FBS and serum for the ex vivo expansion of MSCs which, in the presence of PL, maintain their differentiation potential, immune-phenotype, and immunomodulatory activities [9, 17–19]. In addition to the major role platelets play in hemostasis, they are a principal source of growth factors such as platelet-derived growth factor (PDGF), transforming growth factor (TGF)-β1, vascular endothelial growth factor (VEGF), epidermal growth factor (EGF), attachment factors, and enzymes found in serum. These factors can enhance the recruitment, proliferation, and differentiation of MSCs, but also exhibit anti-inflammatory and angiogenic properties [20–22].

In veterinary medicine, equine PL (ePL) obtained from whole blood via two-step centrifugation can be used instead of FBS for the culture of equine bone marrow-derived MSCs and the short-term expansion of equine cord blood MSCs [6, 23]. Differences in the preparation process of PL such as platelet separation methods (apheresis versus two-step centrifugation), platelet activation (freeze/thaw cycles versus calcium chloride), and removal of platelet fragments, can affect PL growth factor concentrations and therefore influence the proliferative rate and the differentiation capacity of MSCs [5, 24, 25]. We have recently shown that ePL can be safely generated after performing plateletpheresis in awake and standing horses [26]. Furthermore, our studies suggested that ePL suppresses the release of proinflammatory cytokines from LPS-stimulated equine monocytes and that it can be successfully used as a media supplement for the culture of cells without triggering immune responses [27]. There are, however, no detailed long-term MSC functionality studies evaluating the use of ePL obtained from platelet concentrates via plateletpheresis as a media supplement for the ex vivo culture of equine bone marrow-derived MSCs.

Therefore, the main objective of this study was to evaluate the use of ePL pooled from donor horses as a homologous media supplement to rapidly expand equine bone marrow-derived MSCs in culture. As part of the evaluation, we wanted to compare the phenotypic characteristics, trilineage differentiation, and immunomodulatory properties of equine MSCs cultured in ePL compared to MSCs cultured in FBS. We hypothesized that ePL, produced via apheresis, could function as the sole media supplement to culture expand equine bone marrow-derived MSCs in a manner comparable to FBS and without altering their phenotype, trilineage differentiation, or immunomodulatory capacities.

## Methods

### Preparation of ePL

The preparation of ePL was conducted as previously described [26]. Briefly, platelet concentrates were obtained following plateletpheresis (COBE Spectra Dual-Needle) performed in five mix-breed horses belonging to the University of Georgia equine blood donors. The study protocol (IACUC approval #A2015 02–023-Y1-A1) was approved by the University of Georgia Institutional Animal Care and Committee. The platelets were fractured using two freeze-thaw cycles followed by three centrifugation cycles. The ePL was then filtered through a 40-μm Falcon strainer (Corning Inc., Corning, New York) and a 0.45-μm cellulose acetate membrane (EMD Millipore, Billerica, Massachusetts) to remove cellular debris. An equal portion of lysates from each horse was combined to obtain a pooled product after thawing at 37 °C, thorough mixing, and centrifugation at 3485 g for 10 min at 4 °C [28].

### Isolation and culture of equine bone marrow-derived MSCs with FBS or ePL media supplement

Bone marrow was obtained from three healthy mix-breed horses ranging from 3 to 20 years old and cultured under standard conditions. Specifically, bone marrow was aseptically harvested from the sternum of three horses using a bone marrow collection device (Jamshidi, Jorgensen Laboratories, Inc., Loveland, CO) and 15 ml of marrow was aspirated in two syringes containing 2500 units of heparin (Hospira, West-Wards, Eatontown, NJ). Bone marrow-derived MSCs were expanded according to standardized plate adherence techniques [29, 30]. Bone marrow aspirates from each horse were mixed thoroughly and plated equally in two 150-mm culture dishes (TPP, Trasadingen, Switzerland). MSC basal media, containing low-glucose Dulbecco’s modified Eagle’s medium with 4.5% g/L glucose and sodium pyruvate without l-glutamine (DMEM; Cellgro, Mediatech Inc., Manassas, VA), 2 mM l-glutamine (Gibco, Invitrogen, Auckland, New Zealand), 50 U/ml penicillin (Gibco, Invitrogen), 50 μg/ml streptomycin (Gibco, Invitrogen) and 10% FBS was added to each plate and MSCs were cultured under standard conditions (37 °C and 5% CO_2_). Bone marrow was replated and fresh standard cell culture media was added every 3 days until the formation of adherent bone marrow MSC colonies was observed. Upon reaching 80–90% confluency, the cells were harvested with 0.05% trypsin-EDTA (Gibco, Invitrogen), counted using a hemocytometer, and cryopreserved. MSCs were thawed and reseeded as “Passage 1” (P1) at a density of 6000 cells/cm^2^ in the presence of standard cell culture media and allowed to recover. Upon reaching 80–90% confluency, the cells were passaged via digestion with 0.05% trypsin-EDTA (Gibco, Invitrogen) and counted with an automated cell counter (Bio-Rad, Hercules, CA). Experimental cell lines were established by plating MSCs (P2; *n* = 3) at a density of 6000 cells/cm^2^ in 150-mm culture dishes with MSC basal media supplemented with either 10% FBS (FBS culture media) or 10% ePL (ePL culture media). Heparin (2 IU/ml) was added to the ePL culture media to prevent in vitro gel formation. Cells were incubated at 37 °C with 5% CO_2_ and media were replaced every 2 days. For the subsequent passages cells upon reaching 80% confluence were imaged with inverted microscope, passaged, replated, and cryopreserved with either FBS or ePL culture media containing 10% DMSO for future use.

### Cell growth kinetics: population doublings and doubling time

For long-term cell proliferation studies, MSCs from three individual horses (P4; *n* = 3) were plated in triplicate at a density of 1000 cells/cm^2^ in six-well culture plates (Corning™ Costar™, Thermo Scientific, Hampton, NH) with 10% FBS or 10% ePL culture media and permitted to grow under standard cell culture conditions for 32 days. Every 4 days, MSCs in each media formulation were harvested via digestion with 0.05% trypsin and counted via an automatic cell counter (Bio Rad Laboratories, Hercules, CA). Population doublings (PD) and doubling time (DT) for each passage was calculated using the following two formulae [31]:

$$ PD=\mathit{\ln}\;{N}_f/{N}_i/\mathit{\ln}2 $$

$$ DT= CT/ PD $$

where DT is the doubling time in days, CT is the cell culture time, PD is the population doublings, N_*f*_ is the final number of cells, and N_*i*_ is the initial number of cells. All counts were performed in triplicate.

### Cell viability

Cell viability was assessed both with the trypan blue exclusion test and Live/Dead flow cytometry. For the flow cytometry analysis, MSCs in each media formulation were harvested at P5 via digestion with 0.05% trypsin and transferred into a 50-ml conical tube for centrifugation at 200 g for 4 min at room temperature. Following aspiration of excess media, cells were either washed three times with phosphate-buffered saline (PBS) with calcium and magnesium(+/+) and PBS without calcium and magnesium (−/−) or once with PBS (−/−) followed each time by a centrifugation cycle. MSCs were counted using an automated cell counter and stained with 0.4% Trypan blue solution (VWR, Radnor, PA). One million MSCs cultured in FBS or ePL culture media were resuspended in 1 ml PBS and stained with 4 μM ethidium homodimer (Biotium, Fremont, CA) and 2 μM Calcein Blue AM (Thermo Fisher Scientific, Waltman, MA). MSCs stained with either ethidium homodimer or Calcein Blue AM alone were used as control groups. As a negative control, MSCs were harvested, fixed with 4% paraformaldehyde (PFA) for 20 min on ice, washed with PBS, and stained with both ethidium homodimer and Calcein Blue AM. Samples were analyzed by flow cytometry and 50,000 events were collected per sample. Data were analyzed by Flow Jo software (NIH).

### Trilineage differentiation assays

To ensure that equine MSCs cultured in ePL were capable of trilineage differentiation, MSCs at P5 or P6 (*n* = 3), expanded with FBS or ePL culture media, were used for differentiation assays. Undifferentiated MSCs, cultured under standard cell culture conditions, were used as negative controls in all experiments. All experiments were performed in triplicate for each biological replicate.

#### Osteogenesis

Equine MSCs (*n* = 3) were plated at 100,000 cells/well in six-well plates in FBS or ePL culture media until reaching 90% confluency. Cell culture medium was replaced by HyClone AdvanceSTEM osteogenic medium supplemented with 50 μg/ml streptomycin and 50 U/ml penicillin, exchanged every 2–3 days for 28 days. Osteocytes were identified using Van Kossa staining. Specifically, cultures were fixed with 4% PFA on ice for 15 min and stained with 1% silver nitrate for 20 min under ultraviolet light. Plates were washed with distilled water, and unreacted silver was removed by the addition of 5% sodium thiosulfate for 5 min at room temperature. The plates were then washed again, and cultures were imaged using a Leica inverted microscope [32].

For the quantification of calcium deposition, equine MSCs (*n* = 3) were plated at 21,000 cells/cm^2^ in a flat bottom 96-well plate and cultured with media supplemented with FBS or ePL. Upon reaching 90% confluency, cell culture medium was replaced by HyClone AdvanceSTEM osteogenic medium supplemented with 50 μg/ml streptomycin and 50 U/ml penicillin, exchanged every 2–3 days for 28 days. Differentiation of MSCs to osteocytes was determined using the Calcium Liquicolor® Test (StanBio) according the instructions of the manufacturer. Calcium was extracted by the addition of 0.6 N HCL, stored overnight at 4°C; supernatants were combined at a ratio of 1:20 with an equal portion mixture of the color and the base reagent and plates were read at 550 nm (SpectraMax).

#### Adipogenesis

Equine MSCs (*n* = 3) were plated at 100,000 cells/well in six-well plates and cultured with FBS or ePL culture media until reaching 90% confluency. Medium was then replaced by adipogenic media consisting of DMEM, 10% FBS, 5% rabbit serum, 0.5 μΜ dexamethasone, 60 μΜ indomethacin, 0.5 mM IBMX, 1 μM insulin, and 50 U/ml penicillin and 50 μg/ml streptomycin [31]. Medium was exchanged every 2–3 days for 21 days. Cultures were fixed with 4% PFA for 15 min over ice and rinsed with 60% isopropanol. For the identification of lipid droplets an Oil Red O (Sigma, St. Louis, MO) working solution in 60% isopropanol was added to the cultures for 20 min at room temperature. Cultures were imaged using a Leica inverted microscope.

#### Chondrogenesis

One million equine MSCs (*n* = 3) cultured with FBS or ePL culture media were pelleted in sterile polypropylene 15-ml centrifuge tubes and incubated for 48 h with their respective media. The medium was then discharged and replaced with HyClone AdvanceSTEM chondrogenic medium supplemented with 50 μg/ml streptomycin and 50 U/ml penicillin with a medium change every 2–3 days for 28 days. Cultures were fixed with 4% PFA for 15 min over ice, rinsed with PBS, and submitted to histology for staining with Alcian Blue 8GX. Samples were visualized using an Olympus microscope.

For quantification of Alcian Blue staining, equine MSCs (*n* = 3) were plated at 100,000 cells/well in conical bottomed 96-well plates and centrifuged for 10 min; they remained in the presence of ePL or FBS media for 48 h. Cell culture medium was replenished with HyClone AdvanceSTEM chondrogenic medium every 2–3 days for 28 days. A 0.2% Alcian Blue 8GX in 0.1 M HCL solution was applied to the fixed chondrogenic pellets and incubated overnight at room temperature. Pellets were rinsed with PBS and Alcian Blue stain was extracted by the addition of 6 M guanidine/HCL for 24 h at 4 °C; absorbance was measured at 650 nm (Biotek Synergy).

### Phenotypic analysis

The impact of ePL medium on MSC surface molecule expression levels was evaluated by immunophenotypic analysis of MSCs (*n* = 3; P4) expanded with FBS or ePL cell culture media for the expression levels of CD44, CD90, CD105, CD45, and MHC-II markers using flow cytometry. MSCs were harvested, washed three times with PBS by centrifugation at 200 × g for 4 min and fixed with 4% PFA for 15 min over ice. Following three more washes with PBS, cells were pelleted and a blocking solution (10% goat serum (Sigma-Aldrich, St. Louis, MO) diluted in PBS) was added at a final concentration of 1 × 10^6^ cells/ml for 45 min at room temperature. The antibodies used are listed in Table 1. All antibodies used in this study were validated in equine fibroblasts and peripheral blood mononuclear cells (PBMCs).

**Table 1:** List of primary unconjugated antibody characteristics

Marker	Clone	Host	Species reactivity	Dilution	Source
CD44	BAT31A	Mouse	Equine	1:100	VMRD^a^
CD90	5E10	Mouse	Multiple species	1:1000	Biolegend
CD105	SN6	Mouse	Equine	1:250	Bio-Rad
CD45RB	DH16A	Mouse	Equine	1:50	VMRD^a^
MHC II	EqT2	Mouse	Equine	1:200	VMRD^a^

^a^VMRD antibodies are available from the Washington State University Monoclonal Antibody Center

Aliquots of 200 μl containing 2 × 10^5^ cells were centrifuged at 200 × g for 4 min to obtain a dry pellet. After decanting the supernatant, 100 μl of primary unconjugated antibody diluted in blocking solution was added for 1 h at room temperature. Next, samples were washed three times with blocking solution and a secondary fluorescent-conjugated goat anti-mouse IgG antibody (FITC, Sigma-Aldrich, St. Louis, MO) or fluorescent goat anti-mouse IgM antibody (FITC, Sigma-Aldrich, St. Louis, MO) was added to the samples and allowed to further incubate for 1 h at room temperature. Cells were washed three times with blocking solution by centrifugation. MSCs from all animals expanded in FBS or ePL culture media stained with only fluorescence-conjugated secondary antibody were used as control groups for the detection of background autofluorescence staining. To identify nonspecific fluorescence staining, MSCs were stained with unconjugated mouse IgG1 K isotype (1:500, Biolegend, San Diego, CA) or mouse IgM K isotype (1:500, Washington State University Monoclonal Antibody Center, Pullman, WA) followed by the addition of the corresponding fluorescence-conjugated secondary antibody.

Samples were reconstituted in 200 μl blocking buffer, analyzed by flow cytometry (BD Accuri™ C6), and 10,000 events were collected per sample. The mean percentage of positive cells was calculated by subtracting the percentage of positive cells of the fluorescence-conjugated secondary antibody from the percentage of each cell surface marker.

### Effect of MSCs on LPS-driven monocyte activation

Equine PBMCs were isolated according to validated protocols [33]. Briefly, 120 ml of peripheral blood was obtained in two 60-ml syringes each containing 1.5 ml 100 μM EDTA. The leukocyte rich plasma was layered onto leukocyte separation media (Corning Cellgro® Inc., Manassas, VA) and centrifuged for 30 min. PBMCs were collected and viability was assessed via trypan blue exclusion (> 95% for all three biologicals). Cells were resuspended in media consisting of RPMI-1640 supplemented with 10% donor horse serum (DHS) and plated in 150-mm plates for 2 h at 37 °C under 5% CO_2._ After 2 h of incubation, adherent PBMCs, now mainly monocytes [34], were harvested and used for the following experiments.

To assess the immunomodulatory ability of MSCs cultured with either 10% FBS or 10% ePL culture media, a cytokine production assay was performed according to established protocols [35]. Equine MSCs (*n* = 3) at P6 were placed at the bottom of 12-well transwell plates at 100,000 cells/well and allowed to adhere for 12 h in the presence of their corresponding medium. The following day, medium was aspirated and replaced with 1.5 ml of RPMI-1640 supplemented with 10% DHS. Half a milliliter containing 400,000 equine monocytes (ratio 1:4) from the three individuals (*n* = 3) were added to each insert of a transwell plate with pore size of inserts 0.4 μm (Corning, NY), stimulated with 50 ng/ml of *E. coli* 0111:B4 LPS (List Biologicals Inc.) and incubated at 37 °C under 5% CO_2_. At 6 and 18 h time points, cell culture supernatants were collected and assayed for the production of the proinflammatory cytokine tumor necrosis factor (TNF)-α as an indicator of inflammatory responses [27].

### Statistical analysis

Normality of the data was evaluated by visual examination of histograms of the residuals, normal plots of residuals, and by using the Shapiro-Wilks test. Equality of variances was assessed using Levene’s test and plotting residuals against the fitted value. Statistically significant differences for viability, semiquantification of osteogenesis and chondrogenesis, and immunophenotypic profile of MSCs cultured in FBS or ePL were detected by paired *t* test. A mixed model or a two-way repeated-measures analysis of variance (ANOVA) was used to assess the effect of the medium on MSC proliferation and immunomodulatory capacity, respectively. Multiple pairwise comparisons, if necessary, were obtained using the Tukey-Kramer test or Sidak test. All data were analyzed by commercially available statistical packages (Stata version 13.1, StataCorp LP, College Station, TX, or GraphPad Prism 7.0c, La Jolla, CA). The level of significance was set at *P* < 0.05. All results are reported as mean ± standard deviation (SD) unless otherwise stated.

## Results

### Cell growth kinetics: PD and DT

**Fig. 1:**
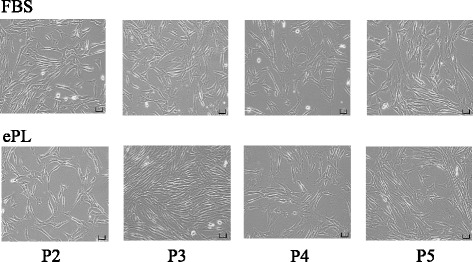
Cellular morphology of equine bone marrow-derived MSCs cultured in 10% fetal bovine serum (FBS) or equine platelet lysate (ePL) culture media from passage P2 to P5. Images are shown from one representative cell line. Scale bars = 100 μm

Cells in both media conditions exhibited similar morphology at every passage, showing spindle-shape characteristics (Fig. 1). These findings were observed in all cell lines and were consistent among triplicates.

**Fig. 2:**
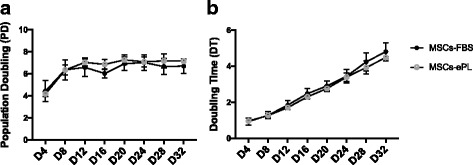
Cell growth kinetics of equine bone marrow-derived mesenchymal stem cells (MSCs) grown in 10% fetal bovine serum (FBS) or equine platelet lysate (ePL) culture media from day 4 (D4) to day 32 (D32). **a** Population doublings (PD) and **b**) doubling time (DT) in days of cells cultured with FBS (MSCs-FBS) or with ePL (MSCs-ePL). Data are shown as mean ± standard deviation; *n* = 3. All data were combined from triplicate cell cultures

A statistically significant difference in proliferation rates for equine MSCs cultured in ePL or FBS at different time points was not identified (*P* > 0.05). Specifically, the PD for MSCs cultured in ePL was 4.17 ± 0.25 at day 4, 6.35 ± 0.49 at day 8, 7.04 ± 0.32 at day 12, 6.97 ± 0.4 at day 16, 7.26 ± 0.43 at day 20, 7.09 ± 0.47 at day 24, 7.17 ± 0.63 at day 28, and 7.14 ± 0.11 at day 32, whereas PD for MSCs cultured in FBS was 4.43 ± 0.95 at day 4, 6.36 ± 0.92 at day 8, 6.58 ± 0.84 at day 12, 6.51± 0.71 at day 16, 6.91 ± 0.62 at day 20, 7.02 ± 0.72 at day 24, 6.66 ± 0.73 at day 28 and 6.70 ± 0.66 at day 32 (Fig. 2a). Moreover, DT (in days) for MSCs in ePL was 0.96 ± 0.1 at day 4, 1.27 ± 0.1 at day 8, 1.7 ± 0.08 at day 12, 2.3 ± 0.13 at day 16, 2.76 ± 0.16 at day 20, 3.4 ± 0.23 at day 24, 3.93 ± 0.37 at day 28, and 4.48 ± 0.07 at day 32, while DT for the FBS control group was 0.93 ± 0.2 at day 4, 1.28 ± 0.1 at day 8, 1.85 ± 0.24 at day 12, 2.48 ± 0.27 at day 16, 2.9 ± 0.28 at day 20, 3.44± 0.37 at day 24, 4.24 ± 0.50 at day 28, and 4.80 ± 0.5 at day 32 (Fig. 2b).

### Cell viability

**Fig. 3:**
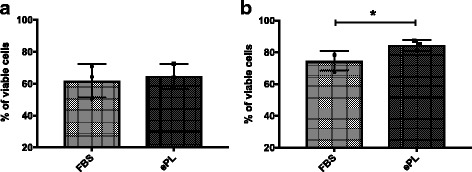
Flow cytometric analysis of the viability of MSCs cultured with different media formulations. The percentage of viable cells cultured with fetal bovine serum (FBS) or equine platelet lysate (ePL) media supplement following (**a**) extensive washing steps or (**b**) a single washing step. **a** Regardless of the media used, there was no statistically significant difference in the percentage of viable cells between FBS and ePL following extensive washing steps. **b** Culture of MSCs with ePL medium resulted in a statistically significant higher percentage of viable cells compared with those in FBS following a single washing step. Data are shown as mean ± standard deviation; *n* = 3. *P < 0.05

MSCs cultured with ePL culture medium exhibited similar percentages of viable cells (64.6 ± 7.67) compared with MSCs cultured in FBS culture medium (61.83 ± 10.42), as evaluated by flow cytometry following extensive washes with PBS (Fig. 3a). The percentage of dead cells in the negative control was 98.1% (data not shown). A decreased percentage of viable MSCs was noticed when cells underwent extensive washes compared to the baseline trypan blue viability assessment (data not shown) regardless of the culture medium used. We chose to perform extensive washes after collecting the cells from the plate in order to mimic the conditions that are commonly used in preparation for the clinical use of MSCs. After noticing a decline in the recovery of viable cells after extensive washes, we chose to include flow cytometry viability data from MSCs collected after a single washing step. Our data revealed that MSCs in ePL had a statistically significant higher percentage of viable cells (84.33 ± 3.45) compared to those in FBS (74.73 ± 6.18) (Fig. 3b).

### Trilineage differentiation assays

The in vitro differentiation assays were performed in equine bone marrow-derived MSCs (*n* = 3) cultured in FBS or ePL culture media at P5 or P6 following their culture in the appropriate differentiation medium. Undifferentiated MSCs cultured for the same period were used as negative controls and failed to differentiate as indicated by lack of specific stain uptake and alteration of cellular morphology.

**Fig. 4:**
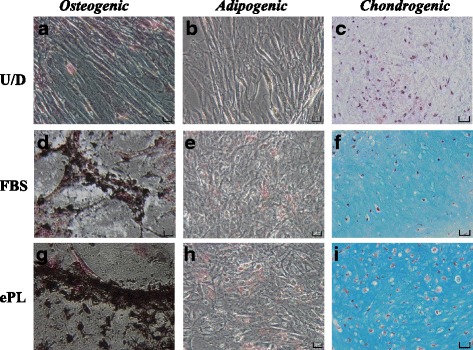
Trilineage differentiation capacity of MSCs (*n* = 3) grown in fetal bovine serum (FBS) or equine platelet lysate (ePL) media supplement. **a** Van Kossa, **b** Oil Red O, and **c** Alcian Blue staining of control undifferentiated cells (U/D). **d** Van Kossa, **e** Oil Red O, and **f** Alcian Blue staining of MSCs previously cultured with FBS media. **g** Van Kossa, **h** Oil Red O, and **i** Alcian Blue staining of MSCs cultured with ePL media. MSCs cultured in both types of media were able to undergo trilineage differentiation. Images are shown from one representative cell line. Scale bars = 100 μm

**Fig. 5:**
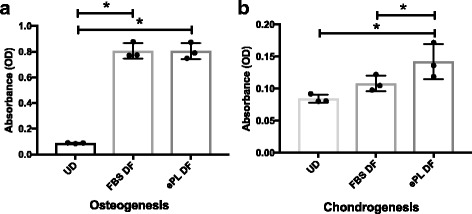
Quantification data of **a** osteogenic and **b** chondrogenic capacity of equine bone marrow-derived MSCs previously cultured in fetal bovine serum (FBS) or equine platelet lysate (ePL). Undifferentiated (UD) MSCs were used as negative control. **a** No statistically significant differences were detected in the amount of calcium deposition during osteogenic differentiation of MSCs grown in ePL (ePL DF) compared with FBS (FBS DF). **b** Statistically significantly elevated levels of proteoglycans were found for MSCs previously grown in ePL (ePL DF) compared with FBS (FBS DF) during chondrogenic differentiation. Data are shown as mean ± standard deviation; *n* = 3. *P < 0.05. OD optical density

Our assays revealed that MSCs from all three cell lines cultured in FBS or ePL media were able to differentiate towards all three lineages following exposure to the corresponding induction medium (Fig. 4). Specifically, MSCs cultured in both media differentiated into osteocytes as shown by increased Van Kossa silver staining for calcium deposition following 28 days of osteogenic induction compared with the undifferentiated group (Fig. 4a, d, g). Our quantification data revealed no statistically significant differences in the amount of calcium production for MSCs grown in FBS (0.81 ± 0.06 OD) compared with ePL (0.80 ± 0.06 OD) (Fig. 5a). For adipogenesis, MSCs cultured in both media differentiated to adipocytes 21 days following induction compared with the undifferentiated cells as indicated by Oil Red O staining for the deposition of lipid droplets (Fig. 4b, e, h). Finally, MSCs in FBS or ePL, following 28 days of chondrogenic media exposure, showed increased proteoglycans by Alcian Blue staining compared with the undifferentiated group (Fig. 4c, f, i). MSCs cultured with ePL exhibited a statistically significantly higher amount of proteoglycan staining (0.14 ± 0.03 OD) as indicated by quantification of Alcian Blue uptake in cell pellets compared with those cultures in FBS (0.11 ± 0.01 OD) (Fig. 5b).

### Phenotypic analysis

The results of the phenotypic analysis are shown in Table 2 as analyzed by flow cytometry for the expression levels of the positive markers CD44, CD90, and CD105 and the negative markers CD45 and MHC-II.

**Table 2:** Cell surface marker expression of equine bone marrow-derived mesenchymal stem cells cultured in fetal bovine serum (FBS) or equine platelet lysate (ePL) media supplement by flow cytometry (*n* = 3)

Marker	FBS	ePL	*P* value
CD44	76.0 ± 7.92	69.45 ± 11.12	0.0895
CD90	87.87 ± 3.29	80.08 ± 1.48	0.0199
CD105	95.99 ± 1.59	96.53 ± 1.21	0.4820
CD45RB	32.29 ± 12.58	18.89 ± 12.37	0.0109
MHC II	1.98 ± 1.04	1.04 ± 0.60	0.2949

Data are shown as mean percentages of positive cells ± SD

The percentage of positive cells was calculated by subtracting the percentage of positive cells of the fluorescence-conjugated secondary antibody from the percentage of each cell surface marker

No statistical significance was detected for the expression levels of CD44, CD105, and MHC-II.

The percentage of positive cells for CD45 was 18.89 ± 12.37% in MSCs cultured in ePL compared with 32.29 ± 12.58% in MSCs cultured in FBS, exhibiting a statistically significant reduction (*P* = 0.0109) of the negative marker. However, MSCs in FBS expressed a statistically significantly higher (87.87 ± 3.29% versus 80.08 ± 1.48%) percentage of positive cells for the marker CD90 compared with MSCs in ePL (*P* = 0.0199).

### Effect of MSCs on LPS-driven monocyte activation

**Fig. 6:**
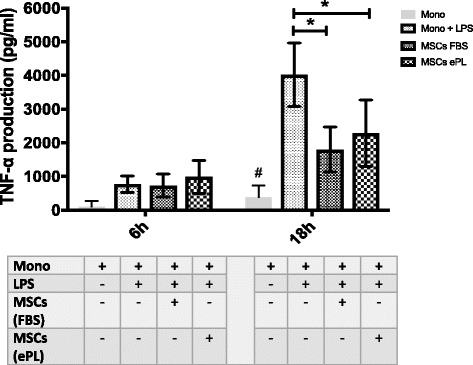
The effect of mesenchymal stem cells (MSCs) cultured in different expansion media on cytokine production from lipopolysaccharide (LPS)-stimulated monocytes. Tumor necrosis factor-α (TNF-α) expression from LPS-stimulated equine monocytes alone or following the addition of MSCs (*n* = 3) cultured in fetal bovine serum (FBS) or equine platelet lysate (ePL) 6 and 18 h following incubation. Unstimulated monocytes (*n* = 3) were used as negative control (Mono). No effect of MSCs was seen on the production levels of TNF-α 6 h following their addition to LPS-stimulated monocytes. A statistically significant decrease in TNF-α was detected when MSCs grown in FBS or ePL were added to LPS-stimulated monocytes 18 h following incubation. Regardless of the expansion media, MSCs retain their ability to modulate the production of proinflammatory cytokines from LPS-stimulated monocytes. Date are shown as mean ± standard deviation; *n* = 3. *P < 0.05 #*P < 0.05*; statistically significant from all other groups

The ability of MSCs to modulate inflammation was tested according to protocols previously validated in our laboratory [35]. After 6 h of incubation with LPS, equine monocytes produced TNF-α concentrations (771.6 ± 246.31 pg/ml) that were markedly greater than those found in the supernatants of the nonstimulated monocytes (100.5 ± 174.04 pg/ml) (Fig. 6). This trend was even more obvious after 18 h when TNF-α concentrations were significantly increased in supernatants from LPS-stimulated monocytes (4025 ± 943.07 pg/ml) compared to nonstimulated controls (382.4 ± 346.98 pg/ml).

No significant reduction in TNF-α production was measured when LPS-stimulated monocytes were coincubated for 6 h with MSCs cultured in either FBS or ePL media (MSCs in FBS, *P* = 0.9999; MSCs in ePL, *P* = 0.9829). In contrast, after 18 h of coculture, MSCs cultured in FBS or ePL media were able to significantly suppress TNF-α production from LPS-stimulated monocytes (*P* = 0.0017 and *P* = 0.0064, respectively) compared with LPS-stimulated monocytes incubated alone. Specifically, LPS-stimulated monocytes alone produced 4025 ± 943.07 pg/ml of TNF-α whereas when MSCs cultured in ePL culture medium were added to LPS-stimulated monocytes, TNF-α production was 2286 ± 983.79 pg/ml. Coculture of LPS-stimulated monocytes with MSCs grown in FBS resulted in the production of 1798 ± 669.75 pg/ml of TNF-α resulting in no significant difference between the suppressive effect of MSCs cultured in ePL or FBS (*P* = 0.6218).

## Discussion

In this study, we were able to show that ePL pooled from donor horses and produced via apheresis can be successfully used as a homologous medium supplement for the in vitro expansion of equine bone marrow-derived MSCs. Moreover, our data support the notion that prolonged culture in ePL medium without FBS preserves the MSC cell surface marker expression and functional characteristics such as trilineage differentiation and immunomodulatory capacity.

One of the major concerns that hampers the clinical application of equine MSCs is the use of FBS for the ex vivo expansion of the cells prior to introduction into the host. Avoiding the use of FBS in cell culture eliminates the concerns related to xeno-immunization of the recipients, transmission of bovine pathogens, and ethical controversies related to the collection methods for FBS [4]. We completed this study because we feel it is of paramount importance to develop MSC cell culture supplements homologous to the species of interest prior to the clinical application of stem cell-based clinical trials or biological therapies. By establishing the use of equine-derived media supplements for the ex vivo expansion of equine MSCs, researchers will be able to proceed to clinical trials without the concerns related to the presence of xenoantigens found in FBS as well as to better standardize an “off-the shelf” stem cell therapeutic product according to international regulations [36–38]. Earlier and recent efforts have focused on the development of xenoprotein-free media for the expansion of MSCs. Studies have shown that the use of serum-free media for the culture of canine and equine MSCs leads to inferior cell proliferation rates and altered immunomodulatory capacity of MSCs compared to standard FBS-based culture media [39]. These findings further prompted the need to develop a homologous supplement rich in growth factors and chemokines that support the proliferation of MSCs and preserve their functional immunomodulatory characteristics.

Only a few studies have investigated the use of ePL produced from whole blood for the expansion of equine MSCs [6, 23]. One of the major advantages of generating PL from platelet concentrates obtained via a standardized plateletpheresis technique is that the final product has a high concentration of platelets with a very low leukocyte contamination [4].

Our data indicate that MSCs cultured in ePL exhibit comparable proliferation rates to those seen with FBS as evaluated by calculation of standard growth kinetic parameters such as PD and DT. It is well documented that culturing human bone marrow- and adipose-derived MSCs with human PL increases the proliferation of MSCs over time compared to FBS [5, 18, 40, 41]. Our results seem to agree with those findings and are comparable to those published in the veterinary literature suggesting that ePL can be used for the expansion of equine MSCs in place of FBS [6, 23]. However, a study conducted by Russell and Koch [6] showed that ePL can be used as a media supplement only for the short-term expansion of equine umbilical cord-derived MSCs. However, the ePL that was used in this study was prepared from whole blood and tested on proliferation abilities of umbilical cord-derived MSCs. As mentioned earlier, differences in the preparation methods of PL used for cell culture and the source of MSCs can affect the proliferative capacity of the cells [5, 24, 25, 42].

It is important to note that our growth kinetic studies showed progressively improved proliferation times the longer that MSCs remained in culture. The fact that significant differences were not detected between MSCs cultured in FBS or ePL at every time point may be attributable to the percentage of ePL we used (10%) for the supplementation of the basal media. Griffiths and colleagues have shown that supplementation of basal media with 5% human PL resulted in a statistically significant increase in the proliferation rates of human MSCs compared to 10% FBS [28]. Future studies should include detailed investigations of escalating concentrations of ePL for the culture of equine MSCs.

Regarding the viability of MSCs following culture expansion, we found interesting yields of cell recovery depending on the methods we used. It is not uncommon for laboratories to include an extensive series of washes with PBS after harvesting MSCs from the culture plates. One of the reasons for this practice is to ensure that cells used in clinical applications do not carry any FBS-derived xenoantigens that would render the cells subject to immune-recognition once introduced to the recipient [43, 44]. When we followed this practice in our experiments and compared pre- and postwash recovery numbers we found a sharp decline in the percentage of viable cells regardless of whether they were cultured in FBS or ePL. In subsequent experiments, we conducted cell viability analyses after only one wash prior to performing viability assays and recorded much higher viability counts similar to those found before washing the cells. Most interestingly, following only one wash MSCs cultured in ePL showed significantly higher viability scores than those cultured in FBS. Based on these results, we suggest that ePL as a homologous medium requires less extensive postculture manipulation resulting in a superior recovery of viable cells.

Trilineage differentiation is one way in which the stem cell research community has attempted to ensure that cultured cells are indeed MSCs [45, 46]. In keeping with this convention, we wanted to verify that MSCs in ePL retained their trilineage differentiation capacity and showed that osteogenic and adipogenic differentiation occurred with no significant differences in cellular morphology compared to MSCs in FBS, in accord with previous studies [5, 9, 41, 47]. With respect to chondrogenic differentiation, we noticed that MSCs in ePL produced statistically significantly greater amounts of proteoglycans which may indicate that ePL promotes a chondrogenic differentiation pattern different to that induced by FBS. This will have profound implications for future clinical applications and especially for the treatment of cartilage defects. The literature has suggested that MSCs grown in the presence of platelet-derived biologicals such as PRP or PL express high levels of chondrogenic markers and extracellular cartilage matrix [48–52], likely because of the release of platelet-derived chondrogenic growth factors such as TGF-β, VEGF, PDGF, insulin-like growth factor (IGF)-1, and fibroblast growth factor (FGF)-2 [53]. TGF-β seems to be especially important for the synthesis of proteoglycans and collagen type II [54, 55] and for the differentiation of MSCs into chondrocytes, a process that has been documented by measuring the chondrogenic-related transcriptional factor Sox9 and mRNA expression of collagen type II [56]. It is possible that the relatively high concentrations of TGF-β present in ePL  [26] might have been responsible for favoring MSC chondrogenic differentiation.

One other criteria that has been proposed as essential by the International Society of Cellular Therapies (ISCT) for the characterization of human MSCs includes the positive identification of the markers CD73, CD90, and CD105, and the absence of CD34, CD45, CD11b or CD14, CD79α or CD19, and HLA class II [46]. Unfortunately, and although much needed, such a consensus has not been reached in equine research regarding the panel of CD markers that should be tested to characterize equine MSCs [57]. Although we believe the veterinary research community should achieve a unanimous opinion on the characterization of MSCs, one obstacle that has hampered this effort is the absence of reliable commercially available monoclonal antibodies specific for equine cells. Regardless, in an attempt to further characterize our cells we evaluated the MSC phenotypic profile by quantifying the expression CD44, CD90, CD105, CD45, and MHC-II, which are markers commonly used in equine stem cell research and have been previously validated in our laboratory. Our immunophenotypic analysis showed that MSCs grown in both media supplements exhibited no statistically significant differences for CD44, CD105, and MHC-II. However, MSCs were characterized by statistically significantly lower percentages of the negative CD45 when cultured in ePL compared to FBS. In addition, for the positive CD90 we saw a decrease in the percentage of positive cells for the MSCs cultured in ePL compared with FBS.

There has been strong evidence suggesting that different types of culture media can affect or even alter the MSC phenotypic profile [58]. In fact, these characteristics can be affected by the isolation techniques and the media used for their culture expansion [59, 60]. Most importantly, it is well documented that contamination of MSC cultures with other cell types is possible, especially when plastic adherence methodologies are used for their initial isolation from bone marrow aspirates resulting in an unexpectedly heterologous cell population [61]. Even though in this study our initial MSC isolation techniques were performed using the plastic adherence method, we were satisfied to find a relatively uniform cell population. It is not unlikely that our isolation technique, although widely applied across laboratories, might be responsible for the increased percentage of MSCs positive for CD45 in the FBS group.

MSCs are clinically attractive because of their reported ability to modulate immune responses and influence inflammatory processes. Specifically, it is well documented that activated MSCs can interact with cells of the immune system such as B cells, T cells, natural killer cells, monocytes/macrophages, dendritic cells, and neutrophils via either direct cell to cell contact or via the expression of soluble factors [62, 63]. Equine monocytes are highly responsive immune cells that are very sensitive to a variety of factors including LPS which stimulates monocytes to secrete proinflammatory cytokines such as TNF-α via a Toll-like receptor (TLR)-mediated pathway. Relevant to our functionality testing, it has been shown that MSCs suppress the activation of LPS-stimulated monocytes and thus the production of proinflammatory cytokines such as TNF-α [35, 64, 65]. We chose to study this immunomodulatory effect as a platform to test differences in TNF-α release between MSCs cultured in FBS or ePL. We conducted these experiments with an established transwell coculture system which allowed us to expose LPS-stimulated monocyte cultures to MSCs grown in FBS or ePL. It is relevant to note that these coculture experiments were conducted in the presence of standard RPMI media appropriate for monocyte proliferation supplemented with 10% DHS. It was important to include DHS because it contains LPS-binding protein (LBP), an essential component for the LPS and TLR4 coupling and the efficient stimulation of monocytes to release their inflammatory payload including TNF-α [66]. Secondly, by only using standard monocyte medium, we ensured that any effect on monocyte activation would likely be due to the MSCs and not the FBS or ePL culture media in which they had been developed.

We found an interesting temporal effect in our experiments noting that MSCs had no effect on TNF-α production following 6 h of coincubation with stimulated monocytes. However, 18 h of coincubation resulted in a significant difference in the expression levels of TNF-α following the addition of MSCs cultured in either FBS or ePL compared with monocyte cultures without MSCs, confirming our hypothesis that MSCs cultured in ePL can modulate inflammation.

A trend noticed when we compared the ability of MSCs to reduce TNF-α production was that those cultured in FBS tended to suppress TNF-α release from LPS-stimulated monocytes more than those cultured in ePL. Studies have suggested that MSCs cultured in human PL, obtained from plateletpheresis products in which 10% of acid citrate dextrose (ACD) was added to donor’s plasma, failed to support their immunomodulatory capacities [67, 68]. Additionally, a detailed study published by Copland and colleagues showed that the presence of fibrinogen in human PL results in an inferior immunosuppressive activity of MSCs compared with those expanded in FBS [69]. In the context of perfecting the processing and manufacturing of our ePL, it may be important to consider collection methods that avoid ACD and consider recovery methods that eliminate fibrinogen from the final product.

## Conclusions

The results of this study provide evidence that ePL can be used instead of FBS for the culture of equine bone marrow-derived MSCs without affecting their characteristics/phenotype and functionality properties. We have shown that ePL medium supplement supports the proliferation and increases the viability of MSCs following a single washing step. Moreover, ePL not only does not impact on the differentiation capacity of MSCs but, according to our data, improves their chondrogenic differentiation potential with profound implications for future clinical applications and especially for the treatment of cartilage defects. MSCs cultured with ePL exhibit comparable immunophenotype and immunomodulatory capacity compared to those in standard cell culture medium. Our results indicate that ePL is an attractive alternative for the ex vivo expansion of equine MSCs before clinical administration, avoiding issues of xeno-immunization related to the use of FBS.

## Abbreviations

ACD: Acid citrate dextrose
DHS: Donor horse serum
DMEM: Dulbecco’s modified Eagle’s medium
DT: Doubling time
ePL: Equine platelet lysate
FBS: Fetal bovine serum
LPS: Lipopolysaccharide
MSC: Mesenchymal stem cell
PBMC: Peripheral blood mononuclear cell
PBS: Phosphate-buffered saline
PD: Population doublings
PDGF: Platelet-derived growth factor
PFA: Paraformaldehyde
PL: Platelet lysate
PRP: Platelet-rich plasma
RPMI: Roswell Park Memorial Institute
TGF: Transforming growth factor
TLR: Toll-like receptor
TNF: Tumor necrosis factor
VEGF: Vascular endothelial growth factor

## References

[1] Glenn JD, Whartenby KA (2014). Mesenchymal stem cells: emerging mechanisms of immunomodulation and therapy. World J Stem Cells.

[2] Farini A, Sitzia C, Erratico S, Meregalli M, Torrente Y. Clinical applications of mesenchymal stem cells in chronic diseases. Stem Cells Int. 2014;2014:1–11. https://www.ncbi.nlm.nih.gov/pubmed/24876848. 10.1155/2014/306573 PMC4021690 24876848

[3] Paul G, Anisimov SV (2013). The secretome of mesenchymal stem cells: potential implications for neuroregeneration. Biochimie.

[4] Burnouf T, Strunk D, Koh MBC, Schallmoser K (2016). Human platelet lysate: replacing fetal bovine serum as a gold standard for human cell propagation?. Biomaterials.

[5] Doucet C, Ernou I, Zhang Y, Llense J-R, Begot L, Holy X, Lataillade J-J (2005). Platelet lysates promote mesenchymal stem cell expansion: a safety substitute for animal serum in cell-based therapy applications. J Cell Physiol.

[6] Russell KA, Koch TG (2016). Equine platelet lysate as an alternative to fetal bovine serum in equine mesenchymal stromal cell culture—too much of a good thing?. Equine Vet J.

[7] Md B, Riccò S, Conti V, Merli E, Ramoni R, Grolli S (2007). Platelet lysate promotes in vitro proliferation of equine mesenchymal stem cells and tenocytes. Vet Res Commun.

[8] Perez-Ilzarbe M, Diez-Campelo M, Aranda P, Tabera S, Lopez T, del Canizo C, Merino J, Moreno C, Andreu EJ, Prosper F, Perez-Simon JA (2009). Comparison of ex vivo expansion culture conditions of mesenchymal stem cells for human cell therapy. Transfusion.

[9] Schallmoser K, Bartmann C, Rohde E, Reinisch A, Kashofer K, Stadelmeyer E, Drexler C, Lanzer G, Linkesch W, Strunk D (2007). Human platelet lysate can replace fetal bovine serum for clinical-scale expansion of functional mesenchymal stromal cells. Transfusion.

[10] Kirikae T, Tamura H, Hashizume M, Kirikae F, Uemura Y, Tanaka S, Yokochi T, Nakano M (1997). Endotoxin contamination in fetal bovine serum and its influence on tumor necrosis factor production by macrophage-like cells J774.1 cultured in the presence of the serum. Int J Immunopharmacol.

[11] Azouna NB, Jenhani F, Regaya Z, Berraeis L, Othman TB, Ducrocq E, Domenech J (2012). Phenotypical and functional characteristics of mesenchymal stem cells from bone marrow: comparison of culture using different media supplemented with human platelet lysate or fetal bovine serum. Stem Cell Res Ther.

[12] Patrikoski M (2014). Different culture conditions modulate the immunological properties of adipose stem cells. Stem Cells Transl Med.

[13] Horwitz EM, Gordon PL, Winston KKK, Marx JC, Neel MD, McNall RY, Muul L, Hofmann T (2002). Isolated allogeneic bone marrow-derived mesenchymal cells engraft and stimulate growth in children with osteogenesis imperfecta: implications for cell therapy of bone. Natl. Acad Sci.

[14] Hemeda H, Giebel B, Wagner W (2014). Evaluation of human platelet lysate versus fetal bovine serum for culture of mesenchymal stromal cells. Cytotherapy.

[15] Even MS, Sandusky CB, Barnard ND (2006). Serum-free hybridoma culture: ethical, scientific and safety considerations. Trends Biotechnol.

[16] Guidance for industry: Guidance for human somatic cell therapy and gene therapy. US Food and Drug Administration, Vaccines, Blood & Biologics; 1998. Available at: https://www.fda.gov/downloads/BiologicsBloodVaccines/GuidanceComplianceRegulatoryInformation/Guidances/CellularandGeneTherapy/UCM081670.pdf.

[17] Castegnaro S, Chieregato K, Maddalena M, Albiero E, Visco C, Madeo D, Pegoraro M, Rodeghiero F (2011). Effect of platelet lysate on the functional and molecular characteristics of mesenchymal stem cells isolated from adipose tissue. Curr Stem Cell Res Ther.

[18] Lange C, Cakiroglu F, Spiess AN, Cappallo-Obermann H, Dierlamm J, Zander AR (2007). Accelerated and safe expansion of human mesenchymal stromal cells in animal serum-free medium for transplantation and regenerative medicine. J Cell Physiol.

[19] Bieback K, Hecker A, Kocaomer A, Lannert H, Schallmoser K, Strunk D, Kluter H (2009). Human alternatives to fetal bovine serum for the expansion of mesenchymal stromal cells from bone marrow. Stem Cells.

[20] Anitua E, Andia I, Sanchez M, Azofra J, del Mar ZM, de la Fuente M, Nurden P, Nurden AT (2005). Autologous preparations rich in growth factors promote proliferation and induce VEGF and HGF production by human tendon cells in culture. J Orthop Res.

[21] Herrmann M, Binder A, Menzel U, Zeiter S, Alini M, Verrier S (2014). CD34/CD133 enriched bone marrow progenitor cells promote neovascularization of tissue engineered constructs in vivo. Stem Cell Res.

[22] Lippross S, Loibl M, Hoppe S, Meury T, Benneker L, Alini M, Verrier S (2011). Platelet released growth factors boost expansion of bone marrow derived CD34(+) and CD133(+) endothelial progenitor cells for autologous grafting. Platelets.

[23] Seo JP, Tsuzuki N, Haneda S, Yamada K, Furuoka H, Tabata Y, Sasaki N (2013). Comparison of allogeneic platelet lysate and fetal bovine serum for in vitro expansion of equine bone marrow-derived mesenchymal stem cells. Res Vet Sci.

[24] Kocaoemer A, Kern S, Kluter H, Bieback K (2007). Human AB serum and thrombin-activated platelet-rich plasma are suitable alternatives to fetal calf serum for the expansion of mesenchymal stem cells from adipose tissue. Stem Cells.

[25] Textor JA, Tablin F (2012). Activation of equine platelet-rich plasma: comparison of methods and characterization of equine autologous thrombin. Vet Surg.

[26] Sumner SM, Naskou MC, Thoresen M, Copland I, Peroni JF (2017). Platelet lysate obtained via plateletpheresis performed in standing and awake equine donors. Transfusion.

[27] Naskou MC, Norton NA, Copland I, Galipeau J, Peroni JF (2017). Innate immune responses of equine monocytes cultured in equine platelet lysate. Vet Immunol Immunopathol.

[28] Griffiths S, Baraniak PR, Copland IB, Nerem RM, McDevitt TC (2013). Human platelet lysate stimulates high-passage and senescent human multipotent mesenchymal stromal cell growth and rejuvenation in vitro. Cytotherapy.

[29] Borjesson DL, Peroni JF (2011). The regenerative medicine laboratory: facilitating stem cell therapy for equine disease. Clin Lab Med.

[30] Mumaw J, Jordan E, Sonnet C, Olabisi R, Olmsted-Davis E, Davis A, Peroni J, West J, West F, Lu Y, Stice S. Rapid heterotrophic ossification with cryopreserved poly(ethylene glycol-)microencapsulated BMP2-expressing MSCs. Int J Biomater. 2012:1–11. 10.1155/2012/861794 PMC3296315 22500171

[31] Vidal MA, Kilroy GE, Johnson JR, Lopez MJ, Moore RM, Gimble JM (2006). Cell growth characteristics and differentiation frequency of adherent equine bone marrow-derived mesenchymal stromal cells: adipogenic and osteogenic capacity. Vet Surg.

[32] Mumaw JL, Schmiedt CW, Breidling S, Sigmund A, Norton NA, Thoreson M, Peroni JF, Hurley DJ (2015). Feline mesenchymal stem cells and supernatant inhibit reactive oxygen species production in cultured feline neutrophils. Res Vet Sci.

[33] Figueiredo MD, Moore JN, Vandenplas ML, Sun WC, Murray TF (2008). Effects of the second-generation synthetic lipid A analogue E5564 on responses to endotoxin in [corrected] equine whole blood and monocytes. Am J Vet Res.

[34] Henry MM, Moore JN (1988). Endotoxin-induced procoagulant activity in equine peripheral blood monocytes. Circ Shock.

[35] Scharf A, Holmes SP, Thoresen M, Mumaw J, Stumpf A, Peroni J (2016). MRI-based assessment of intralesional delivery of bone marrow-derived mesenchymal stem cells in a model of equine tendonitis. Stem Cells Int.

[36] WHO. WHO guidelines on tissue infectivity distribution in transmissible spongiform encephalopathies. Geneva: WHO (World Health Organization); 2006. http://www.who.int/bloodproducts/cs/TSEPUBLISHEDREPORT.pdf?ua=1.

[37] European Medicine Agency. Note for guidance on the use of bovine serum in the manufacture of human biological medicinal products. London: European Medicine Agency; 2003. CPMP/BWP/1793/02 October. http://www.ema.europa.eu/docs/en_GB/document_library/Scientific_guideline/2013/06/WC500143930.pdf.

[38] European Commission. ESAC statement on the use of FCS and other animal-derived supplements. Brussels: European Commission; 2008. https://eurl-ecvam.jrc.ec.europa.eu/about-ecvam/archive-publications/publication/ESAC28_statement_FCS_20080508.pdf.

[39] Clark KC, Kol A, Shahbenderian S, Granick JL, Walker NJ, Borjesson DL (2016). Canine and equine mesenchymal stem cells grown in serum free media have altered immunophenotype. Stem Cell Rev.

[40] Trojahn Kolle SF, Oliveri RS, Glovinski PV, Kirchhoff M, Mathiasen AB, Elberg JJ, Andersen PS, Drzewiecki KT, Fischer-Nielsen A (2013). Pooled human platelet lysate versus fetal bovine serum-investigating the proliferation rate, chromosome stability and angiogenic potential of human adipose tissue-derived stem cells intended for clinical use. Cytotherapy.

[41] Capelli C, Domenghini M, Borleri G, Bellavita P, Poma R, Carobbio A, Mico C, Rambaldi A, Golay J, Introna M (2007). Human platelet lysate allows expansion and clinical grade production of mesenchymal stromal cells from small samples of bone marrow aspirates or marrow filter washouts. Bone Marrow Transplant.

[42] Fazzina R, Iudicone P, Fioravanti D, Bonanno G, Totta P, Zizzari IG, Pierelli L (2016). Potency testing of mesenchymal stromal cell growth expanded in human platelet lysate from different human tissues. Stem Cell Res Ther.

[43] Selvaggi TA, Walker RE, Fleisher TA (1997). Development of antibodies to fetal calf serum with arthus-like reactions in human immunodeficiency virus-infected patients given syngeneic lymphocyte infusions. Blood.

[44] Shih DT, Burnouf T (2015). Preparation, quality criteria, and properties of human blood platelet lysate supplements for ex vivo stem cell expansion. New Biotechnol.

[45] Carrade DD, Borjesson DL (2013). Immunomodulation by mesenchymal stem cells in veterinary species. Comp Med.

[46] Dominici M, Le Blanc K, Mueller I, Slaper-Cortenbach I, Marini F, Krause D, Deans R, Keating A, Prockop D, Horwitz E (2006). Minimal criteria for defining multipotent mesenchymal stromal cells. The International Society for Cellular Therapy position statement. Cytotherapy.

[47] Ben Azouna N, Jenhani F, Regaya Z, Berraeis L, Ben Othman T, Ducrocq E, Domenech J (2012). Phenotypical and functional characteristics of mesenchymal stem cells from bone marrow: comparison of culture using different media supplemented with human platelet lysate or fetal bovine serum. Stem Cell Res Ther.

[48] Gottipamula S, Sharma A, Krishnamurthy S, Majumdar AS, Seetharam RN (2012). Human platelet lysate is an alternative to fetal bovine serum for large-scale expansion of bone marrow-derived mesenchymal stromal cells. Biotechnol Lett.

[49] Mishra A, Tummala P, King A, Lee B, Kraus M, Tse V, Jacobs CR (2009). Buffered platelet-rich plasma enhances mesenchymal stem cell proliferation and chondrogenic differentiation. Tissue Eng Part C Methods.

[50] Prins HJ, Rozemuller H, Vonk-Griffioen S, Verweij VG, Dhert WJ, Slaper-Cortenbach IC, Martens AC (2009). Bone-forming capacity of mesenchymal stromal cells when cultured in the presence of human platelet lysate as substitute for fetal bovine serum. Tissue Eng Part A.

[51] Rubio-Azpeitia E, Andia I (2014). Partnership between platelet-rich plasma and mesenchymal stem cells: in vitro experience. Muscles Ligaments Tendons J.

[52] Shih DT, Chen JC, Chen WY, Kuo YP, Su CY, Burnouf T (2011). Expansion of adipose tissue mesenchymal stromal progenitors in serum-free medium supplemented with virally inactivated allogeneic human platelet lysate. Transfusion.

[53] Kabiri A, Esfandiari E, Esmaeili A, Hashemibeni B, Pourazar A, Mardani M (2014). Platelet-rich plasma application in chondrogenesis. Adv Biomed Res.

[54] Grimaud E, Heymann D, Redini F (2002). Recent advances in TGF-beta effects on chondrocyte metabolism. Potential therapeutic roles of TGF-beta in cartilage disorders. Cytokine Growth Factor Rev.

[55] Fan H, Hu Y, Qin L, Li X, Wu H, Lv R (2006). Porous gelatin-chondroitin-hyaluronate tri-copolymer scaffold containing microspheres loaded with TGF-beta1 induces differentiation of mesenchymal stem cells in vivo for enhancing cartilage repair. J Biomed Mater Res A.

[56] Yu DA, Han J, Kim BS (2012). Stimulation of chondrogenic differentiation of mesenchymal stem cells. Int J Stem Cells.

[57] De Schauwer C, Meyer E, Van de Walle GR, Van Soom A (2011). Markers of stemness in equine mesenchymal stem cells: a plea for uniformity. Theriogenology.

[58] Hagmann S, Moradi B, Frank S, Dreher T, Kammerer PW, Richter W, Gotterbarm T (2013). Different culture media affect growth characteristics, surface marker distribution and chondrogenic differentiation of human bone marrow-derived mesenchymal stromal cells. BMC Musculoskelet Disord.

[59] Kassem M, Kristiansen M, Abdallah BM (2004). Mesenchymal stem cells: cell biology and potential use in therapy. Basic Clin Pharmacol Toxicol.

[60] Herzog EL, Chai L, Krause DS (2003). Plasticity of marrow-derived stem cells. Blood.

[61] Haack-Sorensen M, Friis T, Bindslev L, Mortensen S, Johnsen HE, Kastrup J (2008). Comparison of different culture conditions for human mesenchymal stromal cells for clinical stem cell therapy. Scand J Clin Lab Invest.

[62] Brandau S, Jakob M, Hemeda H, Bruderek K, Janeschik S, Bootz F, Lang S (2010). Tissue-resident mesenchymal stem cells attract peripheral blood neutrophils and enhance their inflammatory activity in response to microbial challenge. J Leukoc Biol.

[63] Peroni JF, Borjesson DL (2011). Anti-inflammatory and immunomodulatory activities of stem cells. Vet Clin North Am Equine Pract.

[64] Lei J, McLane LT, Curtis JE, Temenoff JS (2014). Characterization of a multilayer heparin coating for biomolecule presentation to human mesenchymal stem cell spheroids. Biomater Sci.

[65] Yang Z, Concannon J, Ng KS, Seyb K, Mortensen LJ, Ranganath S, Gu F, Levy O, Tong Z, Martyn K (2016). Tetrandrine identified in a small molecule screen to activate mesenchymal stem cells for enhanced immunomodulation. Sci Rep.

[66] Figueiredo MD, Salter CE, Hurley DJ, Moore JN (2008). A comparison of equine and bovine sera as sources of lipopolysaccharide-binding protein activity in equine monocytes incubated with lipopolysaccharide. Vet Immunol Immunopathol.

[67] Abdelrazik H, Spaggiari GM, Chiossone L, Moretta L (2011). Mesenchymal stem cells expanded in human platelet lysate display a decreased inhibitory capacity on T- and NK-cell proliferation and function. Eur J Immunol.

[68] Bernardo ME, Avanzini MA, Perotti C, Cometa AM, Moretta A, Lenta E, Del Fante C, Novara F, de Silvestri A, Amendola G (2007). Optimization of in vitro expansion of human multipotent mesenchymal stromal cells for cell-therapy approaches: further insights in the search for a fetal calf serum substitute. J Cell Physiol.

[69] Copland IB, Garcia MA, Waller EK, Roback JD, Galipeau J (2013). The effect of platelet lysate fibrinogen on the functionality of MSCs in immunotherapy. Biomaterials.

